# A gene signature in histologically normal surgical margins is predictive of oral carcinoma recurrence

**DOI:** 10.1186/1471-2407-11-437

**Published:** 2011-10-11

**Authors:** Patricia P Reis, Levi Waldron, Bayardo Perez-Ordonez, Melania Pintilie, Natalie Naranjo Galloni, Yali Xuan, Nilva K Cervigne, Giles C Warner, Antti A Makitie, Colleen Simpson, David Goldstein, Dale Brown, Ralph Gilbert, Patrick Gullane, Jonathan Irish, Igor Jurisica, Suzanne Kamel-Reid

**Affiliations:** 1 Div. of Applied Molecular Oncology, Princess Margaret Hospital, Ontario Cancer Institute, University Health Network, Toronto, ON, Canada; 2 Dept. of Surgery and Orthopedics, Faculty of Medicine, São Paulo State University - UNESP, Botucatu, SP, Brazil; 3 Ontario Cancer Institute and the Campbell Family Institute for Cancer Research, Toronto, ON, Canada; 4 Dept. of Pathology, Toronto General Hospital, Ontario Cancer Institute, University Health Network, Toronto, ON, Canada; 5 Dept. of Biostatistics, Princess Margaret Hospital, Ontario Cancer Institute, University Health Network, Toronto, ON, Canada; 6 Dalla Lana School of Public Health Sciences, University of Toronto, Toronto, ON, Canada; 7 Dept. of Otolaryngology, Hospital Calderon Guardia, San Jose, Costa Rica; 8 Dept. of Otolaryngology/Head and Neck Surgery, Worcester Royal Hospital, Worcester, UK; 9 Dept. of Otolaryngology/Head and Neck Surgery, Helsinki University Central Hospital, University of Helsinki, Helsinki, Finland; 10 Dept. of Otolaryngology/Surgical Oncology, Princess Margaret Hospital, Ontario Cancer Institute, University Health Network, Toronto, ON, Canada; 11 Dept. of Computer Science, University of Toronto, Toronto, ON, Canada; 12 Dept. of Laboratory Medicine and Pathobiology, University of Toronto, ON, Canada; 13 Dept. of Medical Biophysics, University of Toronto, Toronto, ON, Canada

**Keywords:** oral squamous cell carcinoma, surgical resection margins, global gene expression profiling, prognostic signature, recurrence

## Abstract

**Background:**

Oral Squamous Cell Carcinoma (OSCC) is a major cause of cancer death worldwide, which is mainly due to recurrence leading to treatment failure and patient death. Histological status of surgical margins is a currently available assessment for recurrence risk in OSCC; however histological status does not predict recurrence, even in patients with histologically negative margins. Therefore, molecular analysis of histologically normal resection margins and the corresponding OSCC may aid in identifying a gene signature predictive of recurrence.

**Methods:**

We used a meta-analysis of 199 samples (OSCCs and normal oral tissues) from five public microarray datasets, in addition to our microarray analysis of 96 OSCCs and histologically normal margins from 24 patients, to train a gene signature for recurrence. Validation was performed by quantitative real-time PCR using 136 samples from an independent cohort of 30 patients.

**Results:**

We identified 138 significantly over-expressed genes (> 2-fold, false discovery rate of 0.01) in OSCC. By penalized likelihood Cox regression, we identified a 4-gene signature with prognostic value for recurrence in our training set. This signature comprised the invasion-related genes *MMP1*, *COL4A1*, *P4HA2*, and *THBS2*. Over-expression of this 4-gene signature in histologically normal margins was associated with recurrence in our training cohort (*p = 0.0003*, logrank test) and in our independent validation cohort (*p = 0.04*, HR = 6.8, logrank test).

**Conclusion:**

Gene expression alterations occur in histologically normal margins in OSCC. Over-expression of the 4-gene signature in histologically normal surgical margins was validated and highly predictive of recurrence in an independent patient cohort. Our findings may be applied to develop a molecular test, which would be clinically useful to help predict which patients are at a higher risk of local recurrence.

## Background

Oral Squamous Cell Carcinoma (OSCC) accounts for 24% of all head and neck cancers [1]. Currently available protocols for treatment of OSCCs include surgery, radiotherapy and chemotherapy. Complete surgical resection is the most important prognostic factor [2], since failure to completely remove a primary tumor is the main cause of patient death. Accuracy of the resection is based on the histological status of the margins, as determined by microscopic evaluation of frozen sections. Presence of epithelial dysplasia or tumor cells in the surgical resection margins is associated with a significant risk (66%) of local recurrence [3]. However, even with histologically normal surgical margins, 10-30% of OSCC patients will still have local recurrence [4], which may lead to treatment failure and patient death.

Since histological status of surgical resection margins alone is not an independent predictor of local recurrence [5], histologically normal margins may harbor underlying genetic changes, which increase the risk of recurrence [6,7]. To date, candidate-gene approach studies have identified genetic alterations in surgical resection margins in head and neck squamous cell carcinoma (HNSCC) from different disease sites, e.g., oral cavity, pharynx/hypopharynx, larynx [6-16]. Genetic alterations identified in HNSCC included over-expression of eIF4E [6,9], TP53 [7,11] and CDKN2A/P16 proteins [7]. Other alterations reported included promoter hypermethylation of *CDKN2A*/*P16 *[13] and *TP53 *mutations [12,16]. In addition, promoter hypermethylation of *CDKN2A*, *CCNAI *and *DCC *was associated with decreased time to head and neck cancer recurrence [10].

Despite all these studies, genetic alterations identified to date have not been used clinically in the assessment of surgical margins, and no study has developed a gene signature that can accurately predict which patients with OSCC are at a higher risk of disease recurrence. This may be due to the lack of studies using high-throughput analysis of multiple surgical margins and matched OSCCs to identify deregulated genes with prognostic value for recurrence. One probable reason for this deficit is that the application of strict inclusion criteria (tumors of a single anatomical site, with all margins being histologically negative, and with multiple margins of adequate RNA quantity and quality for expression analyses), presents a challenge for obtaining adequate sample size to develop a prognostic signature that can predict recurrence in oral carcinoma. We addressed this challenge through a hypothesis-driven approach, based on the hypothesis that commonly over-expressed genes in OSCC and a subset of histologically normal margins are biomarkers of recurrence. We used meta-analysis of published microarray studies to help identify such genes with high confidence, along with global gene expression analysis of histologically normal margins and their corresponding OSCCs in our own study.

## Methods

### Patients

This work was performed with the approval of the University Health Network Research Ethics Board. All patients signed their informed consent before sample collection, and had surgery as their primary treatment. Tissue samples were prospectively collected at time of surgery from the Toronto General Hospital, Toronto, Ontario, Canada. The inclusion criteria of patients were based on primary disease, tumor site (oral cavity), histology of the primary tumor (squamous cell carcinoma), and histological status of surgical resection margins. All patients included in this study had a final pathology report of histologically normal resection margins, meaning none of the patients had a positive margin. An experienced head and neck pathologist (BP-O) performed histological evaluation of all surgical margins (frozen section analysis, as per standard of care) to ensure that they were histologically normal. A section from each tumor sample (OSCC) was stained with hematoxylin-eosin, to confirm the presence of at least 80% tumor cells. The primary tumor, histologically normal margins and a distant normal oral tissue were collected from each patient, in both training and validation cohorts. All tissue samples were snap-frozen in liquid nitrogen until RNA extraction and gene expression analysis. We collected a total of 232 tissue samples from 54 patients (divided into training and validation sets), as described below.

### Samples used for microarrays (training set)

96 samples (histologically normal margins, OSCC and adjacent normal oral tissues) from 24 patients were used for oligonucleotide microarray analysis. Patient clinical data for the training set are shown in Table 1.

**Table 1:** Clinicopathological data, recurrence and outcome data from 24 OSCC patients (N = 96 samples, training set)

Variables	N (%)
**Age (years)**	

Median (range)	58.5 (37-83)

**Gender**	

Male	15 (62.5)

Female	9 (37.5)

**Tobacco use**	

Yes	16 (67)

No	8 (33)

**Alcohol use**	

Yes	17 (71)

No	7 (29)

**Tumor site**	

Tongue	16 (67)

Floor of mouth	4 (17)

Buccal mucosa	2 (8)

Alveolar	2 (8)

**T category**	

T1-T2	12 (50)

T3-T4	12 (50)

**Nodal status**	

Negative (N0)	12 (50)

Positive (N1, N2b, N2c)	12 (50)

**Tumor stage**	

I, II	7 (29)

III, IV	17 (71)

**Tumor grade**	

Moderately differentiated	18 (75)

Poorly differentiated	6 (25)

**Recurrence**	

Yes	9 (37.5)

No	15 (62.5)

**Time to recurrence**	

Median (range)	32 (1.8-34)

**Follow-up**	

Median (range)	13 (1.7-58.8)

**Outcome**	

Alive with no evidence of disease	13 (55)

Alive with disease	2 (8)

Dead of disease	7 (29)

Dead of other causes	2 (8)

### Samples used for quantitative real-time reverse-transcription PCR (RQ-PCR) (validation set)

136 samples (histologically normal margins, OSCC and adjacent normal oral tissues) from an independent cohort of 30 patients were used for RQ-PCR validation analysis. Patient clinical data for the validation set samples are shown in Table 2.

**Table 2:** Clinicopathological data, recurrence data and outcome data from 30 OSCC patients (N = 136 samples, validation set)

Variables	N (%)
**Age (years)**	

Median (range)	67 (48-81)

**Gender**	

Male	21 (70)

Female	9 (30)

**Tobacco use**	

Yes	25 (83)

No	5 (17)

**Alcohol use**	

Yes	21 (70)

No	9 (30)

**Tumor site**	

Tongue	18 (50)

Floor of mouth	8 (27)

Tongue + Floor of mouth	3 (10)

Buccal mucosa	2 (7)

Alveolar	1 (3)

Retromolar	1 (3)

**T category**	

T1-T2	14 (47)

T3-T4	16 (53)

**Nodal status**	

Negative (N0)	24 (80)

Positive (N1, N2b, N2c)	6 (20)

**Tumor stage**	

I, II	13 (43)

III, IV	17 (57)

**Tumor grade**	

Moderately differentiated	23 (77)

Poorly differentiated	7 (23)

**Recurrence**	

Yes	7 (23)

No	23 (77)

**Time to recurrence**	

Median (range)	8 (2-36)

**Follow-up**	

Median (range)	21 (1-81)

**Outcome**	

Alive with no evidence of disease	23 (77)

Alive with disease	4 (13)

Dead of disease	2 (7)

Dead of other causes	1 (3)

### RNA Isolation, Microarrays and Validation Experiments

The detailed protocol for RNA isolation and the experimental details for microarrays using the Human Genome HG-U133A 2.0 plus oligonucleotide arrays (Affymetrix, Santa Clara, CA, USA), as well as validation experiments by RQ-PCR are described in Additional file 1, Methods S1.

### Bioinformatic Analyses

All bioinformatic analyses of array data were performed in the R language and environment for statistical computing (version 2.10.0) implemented on CentOS 5.1 on an IBM HS21 Linux cluster. We pre-processed each public data set using GCRMA normalization [17] with updated Entrez Gene-based chip definition files [18], using the *affy *R package (version 1.24.2) [19]. Genes with evidence of tumor-normal differential expression across all public datasets, with a False Discovery Rate (FDR) of 0.01 and fold-change ≥ 2, were identified using the *rankprod *R package [20]. Gene Ontology enrichment analysis was performed with the *GOstats *R package (version 2.12.0) [21]. We also used GOEAST (Go Enrichment Analysis Software Toolkit) [22] for graphical representation of GO annotations. Analyses are reproducible using the code (available upon request); data have been deposited in NCBI's Gene Expression Omnibus [23] and are accessible through GEO series accession number GSE31056 http://www.ncbi.nlm.nih.gov/geo/query/acc.cgi?acc=GSE31056.

### Meta-analysis of published microarray studies

Our goal was to identify a prognostic signature for recurrence in OSCC, based on the hypothesis that gene expression deregulation occurring in OSCC would be an early indicator of recurrence if gene expression changes are present in a subset of histologically normal surgical resection margins. We performed gene expression profiling of both resection margins and tumors with the purpose of (1) identifying over-expressed genes in tumors as potential markers of recurrence in histologically normal margins, and (2) finding a subset of those genes predictive of recurrence. In order to generate a very high-confidence gene set, we augmented the analysis of our data with a meta-analysis of five published microarray studies [24-28] to reliably identify a set of genes significantly deregulated in OSCC compared to normal oral tissues. These five public data sets were selected based on the availability of raw microarray data, as well as for the inclusion of both oral cavity tumors and either adjacent normal tissues or oral tissues from healthy individuals. Although data from Pyeon et al. [28] included HPV positive and HPV negative head and neck carcinomas from different anatomic sites, we selected oral carcinomas only, which are mainly negative for HPV infection, as shown in a recent study performed by our group [29]. This meta-analysis sample set was composed of a total of 199 samples (141 OSCCs, 38 adjacent normal tissues and 20 healthy normal tissues) from 141 oral cancer patients and 20 healthy individuals (without cancer) (Table 3). We pre-processed data from the different array platforms with updated chip definition files, as described above, to correct outdated probe mapping information from older platforms. We used a Rank Product analysis for the public studies, which considered only the ranking of genes by differential expression between pairs of samples within studies [20,30], avoiding batch and platform-related effects which would occur from directly combining expression values from the different studies. Genes were selected with evidence of up-regulation in tumors with a False Discovery Rate (FDR) of 0.01 and fold-change ≥ 2. We chose to focus on over-expressed genes only, since histologically normal margins may contain only a fraction of genetically altered cells, and the presence of genetically normal cells would likely make down-regulated genes unreliable markers. By using the intersection of genes identified both by meta-analysis and the in-house array training set, we retained only genes that were reproducibly over-expressed compared to normal oral tissues from healthy patients and histologically normal margins. These strict selection criteria for gene signature candidates, based on prior hypothesis, helped to reduce the risk of over-fitting during Cox regression analysis.

**Table 3:** Description of the five publicly available data sets used for meta-analysis

Public Data Set Reference and Accession ID	Sample Size	Array Platform
**Toruner et al. 2004** **GDS1584**	16 oral carcinoma and 4 adjacent normal tissues from 16 patients	Human Genome U133A Plus 2.0 (Affymetrix)

**Ye et al. 2008** **GSE9844**	26 oral carcinoma samples and 12 adjacent normal tissues from 26 patients	Human Genome U133A Plus 2.0 (Affymetrix)

**Kuriakose et al. 2004** **GDS2520**	22 head and neck carcinomas and 22 adjacent normal tissues from 22 patients	Human Genome U95A (Affymetrix)

**Sticht et al. 2008** **GSE10121**	35 oral carcinoma samples from 35 patients and 6 normal oral tissues from healthy individuals	Human Oligo Set 4.0 (Operon)

**Pyeon et al., 2007** **GSE6791**	42 head and neck carcinoma samples from 42 patients and 14 normal oral tissues from healthy individuals	Human Genome U133A Plus 2.0 (Affymetrix)

### Data analysis of in-house microarray study

Microarray results from our study were normalized as described above, along with normal oral tissue samples from healthy individuals also assayed by Affymetrix Human Genome U133 Plus 2.0 oligonucleotide arrays (downloaded from GEO accession number GSE6791). Probesets with low expression (75^th ^percentile below log_2_(100)) or low variance (IQR on log_2 _scale < 0.5) were filtered [31] as well as the quality control probe sets. The *treat *function from LIMMA: Linear Models for Microarray Analysis (version 3.2.1) [32] was used to identify genes ≥ 2-fold up-regulated in tumors compared to margins from our study, with FDR = 0.01. We took the intersection of genes identified in both the meta-analysis and in-house microarray experiment, and this intersection analysis produced a list of 138 genes, which were used as the feature set for developing the gene signature for recurrence.

### Penalized Cox regression

LASSO penalized Cox regression was used to train a predictive risk score from the maximum expression of the 138 genes in any margin of each patient (all these 138 genes being found to be over-expressed in OSCC). Normalized expression values were first converted to Z-scores for each gene, to ensure equal effect of the penalty on all coefficients, as is standard for penalized regression. We applied LASSO penalized Cox regression as implemented in the *penalized *R package (version 0.9-27) [33], to condition a linear risk score with time to recurrence as the event of interest. In keeping with the hypothesis that over-expression of these genes is predictive of recurrence, we constrained the regression coefficients to positive values, and selected the L1 penalty parameter by optimizing 10-fold cross-validated likelihood. Since the cross-validated likelihood depends on the randomized folding of the data, we repeated the regression 50 times and selected the model with the highest cross-validated likelihood.

## Results

### Patient characteristics: OSCC recurrence

As shown in Table 1 and Table 2, 9/24 patients (training set) and 7/30 patients (independent validation set) had disease recurrence. Median time to local recurrence (by Kaplan Meier estimate) of patients in the training set was 32 months (range 2-34 months). Similarly, patients from the validation set recurred within 2-36 months. All patients had local recurrence, and some patients also had regional and/or distant failure (detailed data in Tables 1 and 2). Median (by reverse Kaplan-Meier estimate) and range of follow-up times of patients were 20 months (1.4-57 months) in the training set and 23 months (1-81 months) in the validation set.

### Differentially expressed genes in margins, OSCC and normal oral tissues

**Figure 1:**
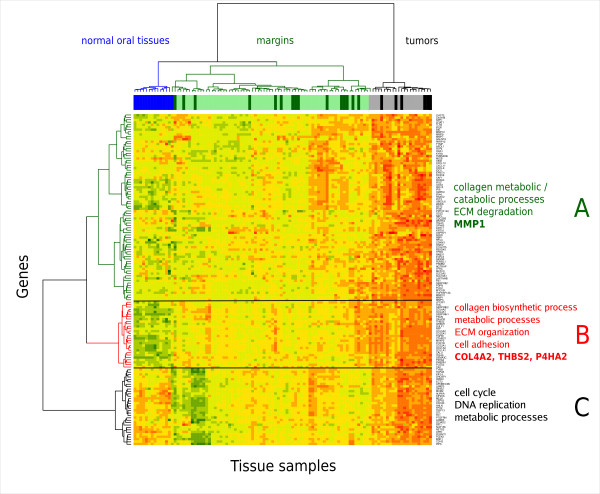
**Heatmap of 138 genes up-regulated in OSCC**. Expression values for each row (gene) are scaled to z-scores for visualization. Margins and tumors annotated with darker colors above the heatmap are from patients who experienced recurrence.

**Figure 2:**
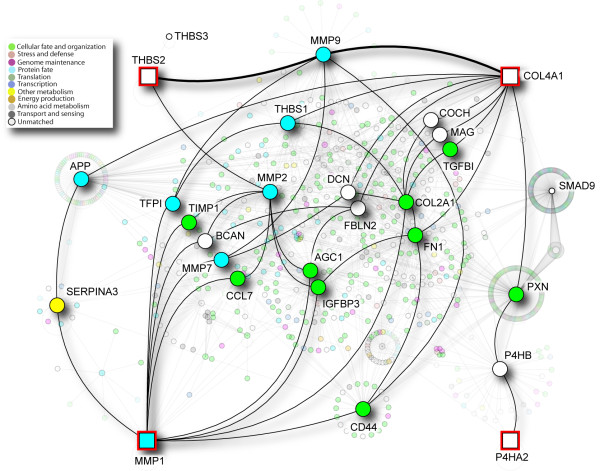
**Protein-protein interaction network of 138 genes**. I2D version 1.72 was used to identify protein interactions for the 138 genes shown in the heatmap. The resulting network was visualized using NAViGaTOR 2.1.14 http://ophid.utoronto.ca/navigator. Color of nodes corresponds to Gene Ontology biological function, as described in the legend. Red-highlighted squares represent the four genes in the signature of OSCC recurrence.

Meta-analysis of the five public data sets (Table 3) identified 667 up-regulated genes in OSCC; results are provided in Additional file 2, Table S1. Requiring two-fold up-regulation in tumors in both the meta-analysis of public datasets and our microarray experiment, with FDR < 0.01, identified 138 over-expressed genes (Additional file 3, Table S2). The expression patterns of these genes in tumors, margins, and normal oral tissue samples from healthy individuals are shown as a heatmap in Figure 1. As seen in the hierarchical clustering, the 138 genes accurately discriminate between the tumors and margins of the in-house study, as well as normal oral tissues from a publicly available study (as described in Methods). We performed LASSO penalized regression of these 138 genes and selected a total of six genes. Of these six genes, we retained four genes with the largest coefficients (*MMP1*, *COL4A1*, *P4HA2 *and *THBS2*), and eliminated two genes with very small coefficients (*PXDN *and *PMEPA1*), which together contributed less than 5% of the magnitude of the risk score. These four genes constituted a 4-gene signature of recurrence. In the heatmap, Gene cluster "B" contains three of the four genes in the signature (*COL4A1*, *P4HA2 *and *THBS2*), and it shows frequent up-regulation in the surgical margins compared to the normal oral tissues. Strikingly, *MMP1*, found in gene cluster "A", shows less frequent over-expression in the margins, but has extreme differential expression between margins and OSCCs (400-fold up-regulation as detected by microarrays, and 800-fold up-regulation, validated by RQ-PCR). The proteins encoded by the 138 genes are also shown in a protein interaction network that highlights the most highly inter-connected proteins (Figure 2 and Additional file 1, Methods S1). In the heatmap, the main features of clusters A and B are the large number of interacting MMP proteins in cluster A, which contains *MMP1*, and collagens plus *TGFB1 *in cluster B, which also contains *P4HA2*, *THBS2 *and *COL4A1 *genes of the signature. The large number of MMPs and collagen proteins are closely connected; in particular, MMP9 interacts with both THBS2 and COL4A1, and indirectly with MMP1.

Results of Gene Ontology (GO) enrichment analysis of all 138 genes are presented in Additional file 4, Table S3. Graphical representations of GO annotations are shown in Additional file 5, GO biological processes, Additional file 6, GO cellular component and Additional file 7, GO molecular function.

### Over-expressed genes in surgical margins: prognostic value for recurrence

We examined these 138 genes (see above) for univariate association with recurrence in the microarray training data, which, since recurrence was not used in the selection of these genes, constitutes the first validation of our study hypothesis. We found significant enrichment for univariate association with recurrence in these 138 genes (see Additional file 8, Figure S1 for comparison of the distribution of nominal p-values with a null distribution empirically determined by permutation of the outcome labels).

### Four-gene prognostic signature for OSCC recurrence

**Figure 3:**
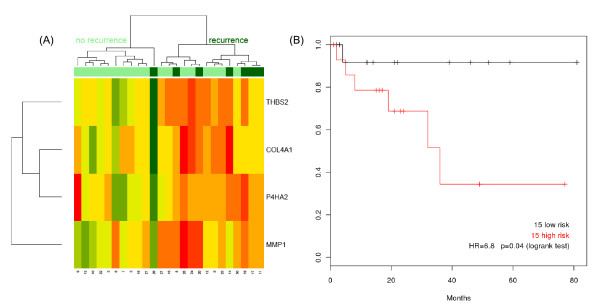
**Heatmap of validation data and Kaplan-Meier plot of disease recurrence**. (A) Unsupervised hierarchical clustering of the quantitative real-time PCR (validation data) showing the maximum expression levels of *MMP1*, *P4HA2*, *THBS2 *and *COL4A1 *in margins from patients with and without recurrence and with a follow-up time ≥ 12 months. Margins annotated with darker green above the heatmap are from patients who experienced recurrence. Margins from patients with locally recurrent tumors show increased expression levels of the four-gene signature compared to patients who did not recur. (B) Kaplan-Meier plot of quantitative real-time PCR data for patients in the validation set. Patients are assigned to high or low risk based on their four-gene signature risk score. As seen in the Kaplan-Meier plot, patients with over-expression of the 4-gene signature are at high risk for disease recurrence; all patients who experienced recurrence in the validation set are in the high risk group, suggesting that over-expression of this signature was highly predictive of recurrence in the validation set.

We performed quantitative PCR validation of the 4-gene signature (*MMP1*, *COL4A1*, *P4HA2 *and *THBS2*) in a separate patient cohort (Table 2). This validation analysis confirmed that all four genes were up-regulated in margins and OSCC samples from patients with disease recurrence compared to margins and OSCCs from patients who did not recur (Figure 3A). The risk score was calculated using the maximum expression in any margin of each patient, and was dichotomized at the median score. These risk groups were predictive of recurrence in an independent test cohort assayed by RQ-PCR (136 samples; N = 30 patients) (HR = 6.8, *p = 0.04*, logrank test) (Figure 3B). In multivariate Cox analysis, correction for T (tumor size) and N (nodal status) resulted only in a slight increase in significance level of the four-gene risk score (*p = 0.06*, likelihood ratio test). Clinical variables, alone or in combination, were not predictive of recurrence in either training or validation cohorts. The coefficients of the 4-gene risk score, for use with Z-score scaled expression values, are summarized in Table 4.

**Table 4:** Coefficients of the linear risk score for z-score normalized log2-expression values.

Gene	Coefficient	FC (microarray)	FC (RQ-PCR)	*p*-value (qPCR)
***MMP1***	0.63	405	798	9E-16

***COL4A1***	0.25	3.7	4.3	7E-09

***P4HA2***	0.45	2.7	2.8	1E-06

***THBS2***	0.34	3	1.9	6E-03

Fold-change (FC) is the geometric-average expression in tumors relative to surgical resection margins. *P*-values are for tumor/margin differential expression in the qPCR (independent validation set) (Wilcoxon Rank Sum test)

In addition, we performed a survival Receiver Operating Characteristic (ROC) analysis to demonstrate the sensitivity and specificity of the model in the test set. This analysis showed the effect of altering the cutoff threshold between high and low-risk patients. Our results showed that the use of a very high threshold to define high-risk patients predicts a majority of recurrences at a low false-positive rate (20%). The area under the ROC curve (AUC) for recurrence within 36 months was 0.73, an improvement over the expected AUC of 0.5 for unpredictive models (Additional file 9, Figure S2). While we maintained the standard median cutoff due to the limited sample size, a larger study in the future may be able to further fine-tune the cutoff threshold to optimize sensitivity and specificity in the context of the relative risks that treatment options informed by this prognostic score entail.

### Effect of reducing the number of available margins

Simulating the selection of only a single margin from each patient, the 4-gene signature maintained a predictive effect in both the training and validation sets (median HR = 2.2 in the training set and 1.8 in the validation set, with 82% and 99% of bootstrapped hazard ratios greater than the no-effect value of HR = 1) (Additional file 10, Figure S3). Results from the bootstrap simulations showed smaller hazard ratios, compared with hazard ratios obtained when using the maximum expression value from several margins. This difference could not be explained by association between the number of margins and recurrence in the training set (HR = 1.17, p = 0.69 logrank test) or the validation set (HR = 1.30, p = 0.24 logrank test). However, a plausible explanation for this difference is that increasing the number of observed margins increases the probability of observing genetic alterations spreading asymmetrically from the tumor, since genetic alterations are not seen in all surgical margins.

## Discussion

It is known that histologically normal margins may harbor genetic changes also found in the primary tumor, as shown by studies in HNSCC, including oral carcinomas [7]. In oral carcinoma, local recurrence may arise from cancer cells left behind after surgery, undetectable by histopathology (minimal residual cancer), or from fields of genetically altered cells with the potential to give rise to a new carcinoma [34]; such fields precede the tumor and can be detected in the surrounding mucosa (surgical resection margins). Molecular changes that are commonly detected in margins as well as the corresponding tumor could indicate that pre-malignant or malignant clones were able to migrate to the surrounding tissue, giving rise to a primary tumor recurrence [35].

Herein, we show that global gene expression analysis of histologically normal margins and OSCC is a valuable approach for the identification of deregulated genes and pathways associated with OSCC recurrence. We used a multi-step procedure including our in-house whole-genome expression profiling experiment and a meta-analysis of five published microarray datasets to develop a 4-gene signature (*MMP1*, *COL4A1*, *THBS2 *and *P4HA2*) for prediction of recurrence in OSCC. This signature is based on genes found to be consistently over-expressed in OSCC as compared to normal oral mucosa; these genes are also over-expressed in a subset of histologically normal surgical resection margins, and their over-expression in such margins provides an indication of the presence of genetic changes before histological alterations can be detected by histology. Notably, our initial analyses reveal that this 4-gene signature predicted recurrence in two of the patients (Pts. 17 and 20, Table 2, validation set) who had not recurred until the latest update of the clinical data for recurrence status. Both of these patients had local recurrence, 8 and 19 months after surgery, respectively.

Genes identified in the 4-gene signature (*MMP1*, *COL4A1*, *THBS2 *and *P4HA2*) play major roles in cell-cell and/or cell-matrix interaction, and invasion. The direct and indirect partners of these genes are illustrated in Figure 2. The functions of two genes (*P4HA2 *and *THBS2*) in our signature of OSCC recurrence and their roles in cancer are not well understood. *P4HA2 *encodes a key enzyme involved in collagen synthesis, and its over-expression has been previously reported in papillary thyroid cancer [36]. *THBS2 *is a matricellular protein that encodes an adhesive glycoprotein and interacts with other proteins to modulate cell-matrix interactions [37]. Interestingly, THBS2 is associated with tumor growth in adult mouse tissues [37]. The two other genes in our OSCC recurrence signature (*COL4A1 *and *MMP1*) are better characterized in cancer. *COL4A1 *encodes the major type IV alpha collagen chain and is one of the main components of basement membranes. Basement membranes have several important biological roles, and are essential for embryonic development, proper tissue architecture, and tissue remodeling [38]. COL4A1 binds other collagens (COL4A2, 3, 4, 5 and 6), as well as LAMC2 (laminin, gamma 2), TGFB1 (transforming growth factor, beta 1), among other proteins (Figure 2) http://www.ihop-net.org, playing a relevant role in extracellular matrix-receptor interaction and focal adhesion [39]. The extracellular matrix undergoes constant remodeling; during this process, proteins such as MMP1 can degrade the extracellular matrix proteins (e.g., collagen IV), and contribute to invasion and metastasis [40]. In cancer, combined over-expression of *COL4A1 *and *LAMC2 *can distinguish OSCC from clinically normal oral cavity/oropharynx tissues [41]; this latter study suggests that *COL4A1 *over-expression may be a useful biomarker for early detection of malignancy.

*MMP1 *belongs to the family of matrix metalloproteases, which are key proteases involved in extracellular matrix (ECM) degradation [42]. *MMP1 *encodes a collagenase, which is secreted by tumor cells as well as by stromal cells stimulated by the tumor; this secreted enzyme is responsible for breaking down interstitial collagens type I, II and III in normal physiological processes (e.g., tissue remodeling) as well as disease processes (e.g., cancer) [42]. It is believed that the mechanism of up-regulation of most of the MMPs is likely due to transcriptional changes, which may occur following alterations in oncogenes and/or tumor suppressor genes [42]. Indeed, MMP1 has been previously reported as consistently over-expressed in oral carcinoma compared to adjacent normal tissues [43-45] and suggested as a biomarker of malignant transformation in precursor lesions in oral cancer [44,46,47]. In HNSCC, over-expression of several genes with roles in invasion and metastasis, including MMPs, were previously associated with treatment failure of HNSCC [48]. In our study, *MMP1 *was over-expressed in a subset of margins exclusively from patients with recurrent OSCC, and showed the highest fold-change of up-regulation in OSCC compared to margins. Our results are in agreement with the literature findings of *MMP1 *over-expression associated with progression of pre-malignant oral lesions; in this context, our data showing *MMP1 *over-expression in histologically normal surgical margins from patients who developed local recurrence support the notion that *MMP1 *may be involved in initial steps of cellular transformation and tumorigenesis, as well as invasion of oral carcinoma cells. Indeed, matrix metalloproteinases play an important role not only in invasion and metastasis but also in early stages of cancer development/progression [42].

Our data suggest that histologically normal surgical resection margins that over-express *MMP1*, *COL4A1*, *THBS2 *and *P4HA2 *are indicative of an increased risk of recurrence in OSCC. Patients at higher risk of recurrence could potentially benefit from closer disease monitoring and/or adjuvant post-operative radiation treatment, even in the absence of other clinical and histopathological indicators, such as advanced disease stage and perineural invasion.

## Conclusion

Considering that our 4-gene signature showed prognostic value for recurrence in two independent patient cohorts, over-expression of this signature may be used for molecular analysis of histologically negative margins in oral carcinoma.

## Competing interests

The authors declare that they have no competing interests.

## Authors' contributions

PPR, BPO, IJ and SKR designed the study. LW, IJ and MP performed bioinformatics and statistical data analyses. PPR, LW, IJ and SKR drafted the manuscript. YX and NKC helped with quantitative PCR experiments and data interpretation. NNG, GCW and AAM helped with study design, identified patients and collected samples. NNG and AAM helped draft the manuscript. NNG, CS, DG, DB, RG, PG and JI helped identify patients, collected samples and provided detailed clinical data of patients. All authors read and approved the final manuscript.

## Pre-publication history

The pre-publication history for this paper can be accessed here:

http://www.biomedcentral.com/1471-2407/11/437/prepub

## Supplementary Material

Additional file 1 **Methods S1**. Description of methods used for RNA isolation, oligonucleotide array experiments, quantitative real-time reverse-transcription PCR validation and protein-protein interaction network analysis. Click here for file

Additional file 2 **Table S1**. Results of meta-analysis of the five public data sets identified 667 up-regulated genes in OSCC. Click here for file

Additional file 3 **Table S2**. List of 138 over-expressed genes, with FDR < 0.01, identified in both the meta-analysis of public datasets and our microarray experiment. Click here for file

Additional file 4 **Table S3**. Results of Gene Ontology enrichment analysis of the 138 genes identified as over-expressed in both the public datasets and our microarray experiment. Click here for file

Additional file 5 **GO biological function**. Graphical representation of GO annotation (biological function). Click here for file

Additional file 6 **GO cellular component**. Graphical representation of GO annotation (cellular component). Click here for file

Additional file 7 **GO molecular function**. Graphical representation of GO annotation (molecular function). Click here for file

Additional file 8 **Figure S1**. Distribution of nominal p-values for univariate association of the 138 genes identified as over-expressed in OSCC with recurrence. P-values were determined by Cox regression using the maximum expression in any margin of each patient. The empirical null distribution was determined from association of these same genes with 1,000 permutations of the outcome labels. The observed nominal p-values are significantly enriched for small values (p = 0.001, Kolmogorov-Smirnov test). It is worth noting that recurrence was not used at any stage in the selection of these genes. Click here for file

Additional file 9 **Figure S2**. Survival Receiver Operating Characteristic curve for recurrence at 36 months in the test set. Using a high threshold to define high-risk patients predicts a majority of recurrences (true positives) at a low false-positive rate (20%). While we maintained the standard median cutoff for this study due to the limited sample size, a larger study in the future may be able to further tune the cutoff threshold to optimize sensitivity and specificity in the context of the relative risks that treatment options informed by this prognostic score entail. The area under the ROC curve (AUC) for recurrence within 36 months is 0.73, which is an improvement over the expected AUC of 0.5 for non-predictive risk scores. Click here for file

Additional file 10 **Figure S3**. Bootstrap validation of four-gene signature risk score in training and validation sets. Density lines represent the distribution of hazard ratios observed in 1,000 re-samplings of a single margin, randomly chosen, from each patient. Click here for file
