# Supplementary material for: A gene signature in histologically normal surgical margins is predictive of oral carcinoma recurrence
Source: BMC Cancer. 2011 Oct 11;11:437. doi: 10.1186/1471-2407-11-437 (PMC3198722; doi:10.1186/1471-2407-11-437)
Supplement: Additional file 1 — Methods S1. Description of methods used for RNA isolation, oligonucleotide array experiments, quantitative real-time reverse-transcription PCR validation and protein-protein interaction network analysis. [file 1471-2407-11-437-S1.DOC]

***RNA Isolation***

Total RNA was extracted from all tissues using Trizol reagent (Life Technologies, Inc., Burlington, ON, Canada), followed by purification using the Qiagen RNeasy kit/DNase RNase-free set (Qiagen, Valencia, CA, USA), according to manufacturer’s instructions. RNA was quantified by spectrophotometry and its quality was assessed using the 2100 Bioanalyzer (Firmware v.A.01.16, Agilent Technologies, Canada). All samples were of sufficient quantity and quality for arrays and quantitative real-time PCR (RQ-PCR) analyses.

***Oligonucleotide Array Experiments***

We used the HG-U133A 2.0 plus oligonucleotide microarrays (Affymetrix, Santa Clara, CA, USA), which contain 40,000 probes representing 20,000 unique human genes. Labeling and hybridization to arrays were performed by The Centre for Applied Genomics, Medical and Related Sciences Centre (MaRS), Toronto, ON, Canada. Briefly, 10 μg of total RNA was used for cRNA amplification using the Invitrogen SuperScript kit (Life Technologies, Inc., Burlington, ON, Canada). Amplification and biotin labeling of antisense cRNA was performed using the Enzo® BioArray^TM^ High Yield^TM^ RNA transcript labeling kit (Enzo Diagnostics, Farmingdale, NY, USA), according to the manufacturer’s instructions. Microarray slides were scanned using the GeneArray 2500 scanner (Agilent Technologies).

***Quantitative real-time PCR (RQ-PCR) validation***

RQ-PCR validation was performed using the 7900 Sequence Detection System and SYBR Green I fluorescent dye (Applied Biosystems, Foster City, CA) as previously described [1, 2]. Primer sequences are available upon request. Reactions were performed in duplicate for each sample and primer set. Dissociation curves were run for all reactions to ensure specificity. RQ-PCR data was normalized by the ΔΔCt method [3], with *GAPDH* as the internal control gene and a commercially available universal normal tongue RNA (Stratagene, Santa Clara, CA) as the reference sample.

***Protein-protein interaction network analysis***

Protein interaction network and pathway analyses were performed using the Interologous Interaction Database (I2D, v 1.71; <http://ophid.utoronto.ca/i2d>) [4]. Network visualization and analysis was done in NAViGaTOR 2.1.15 (<http://ophid.utoronto.ca/navigator>) [5, 6]. GO annotations and KEGG pathways of our data plus the literature data were identified using the Gene Annotation Co-Occurrence Discovery Tool (GeneCODIS) database (<http://genecodis.dacya.ucm.es/>) [7] and the Molecular Signatures Database (MSigDB) (<http://www.broad.mit.edu/gsea/msigdb/index.jsp>) [8].

***References***

1. Reis PP, Rogatto SR, Kowalski LP, Nishimoto IN, Montovani JC, Corpus G, Squire JA, Kamel-Reid S: **Quantitative real-time PCR identifies a critical region of deletion on 22q13 related to prognosis in oral cancer**. *Oncogene* 2002, **21**(42):6480-6487.

2. Reis PPB, RR.; Machado, J.; MacMillan, C.; Pintilie, M.; Sukhai, MA.; Perez-Ordonez, B.; Gullane, P.; Irish, J.; Kamel-Reid, S.: **Claudin 1 over-expression increases invasion and is associated with aggressive histological features in oral squamous cell carcinoma**. *Cancer* 2008.

3. Livak KJ, Schmittgen TD: **Analysis of relative gene expression data using real-time quantitative PCR and the 2(-Delta Delta C(T)) Method**. *Methods* 2001, **25**(4):402-408.

4. Brown KR, Jurisica I: **Online predicted human interaction database**. *Bioinformatics* 2005, **21**(9):2076-2082.

5. Brown KR, Otasek D, Ali M, McGuffin MJ, Xie W, Devani B, Toch IL, Jurisica I: **NAViGaTOR: Network Analysis, Visualization and Graphing Toronto**. *Bioinformatics* 2009, **25**(24):3327-3329.

6. McGuffin MJ, Jurisica I: **Interaction techniques for selecting and manipulating subgraphs in network visualizations**. *IEEE Trans Vis Comput Graph* 2009, **15**(6):937-944.

7. Carmona-Saez P, Chagoyen M, Tirado F, Carazo JM, Pascual-Montano A: **GENECODIS: a web-based tool for finding significant concurrent annotations in gene lists**. *Genome Biol* 2007, **8**(1):R3.

8. Subramanian A, Tamayo P, Mootha VK, Mukherjee S, Ebert BL, Gillette MA, Paulovich A, Pomeroy SL, Golub TR, Lander ES *et al*: **Gene set enrichment analysis: a knowledge-based approach for interpreting genome-wide expression profiles**. *Proc Natl Acad Sci U S A* 2005, **102**(43):15545-15550.
