# Supplementary figures and images for: A gene signature in histologically normal surgical margins is predictive of oral carcinoma recurrence
Source: BMC Cancer. 2011 Oct 11;11:437. doi: 10.1186/1471-2407-11-437 (PMC3198722; doi:10.1186/1471-2407-11-437)

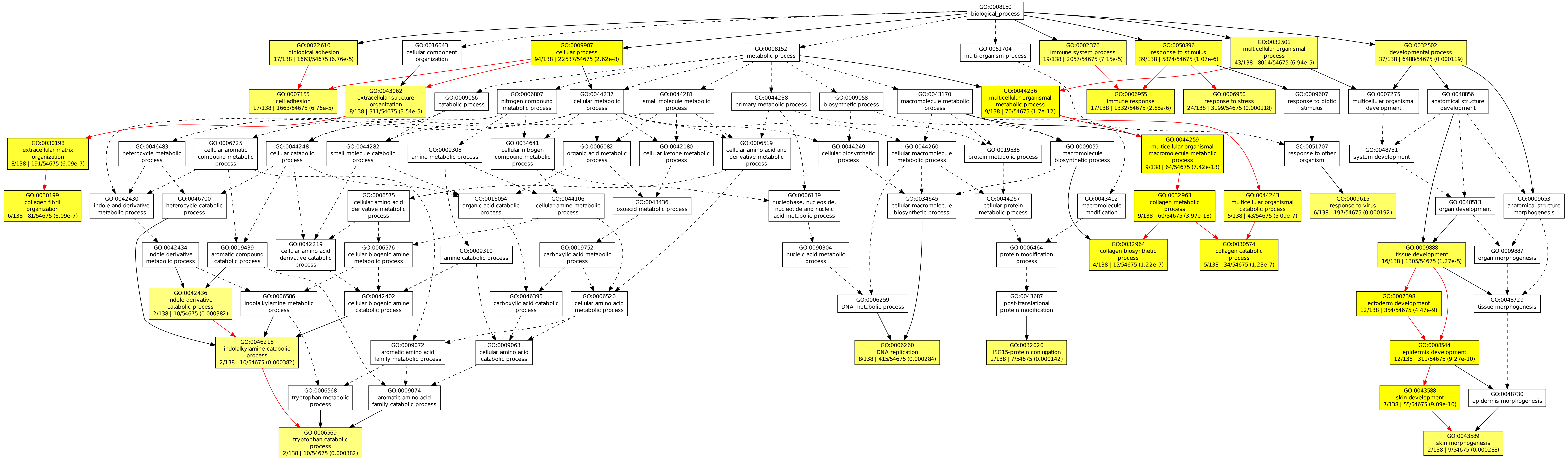

Supplement: Additional file 5 — GO biological function. Graphical representation of GO annotation (biological function). [file 1471-2407-11-437-S5.PDF]

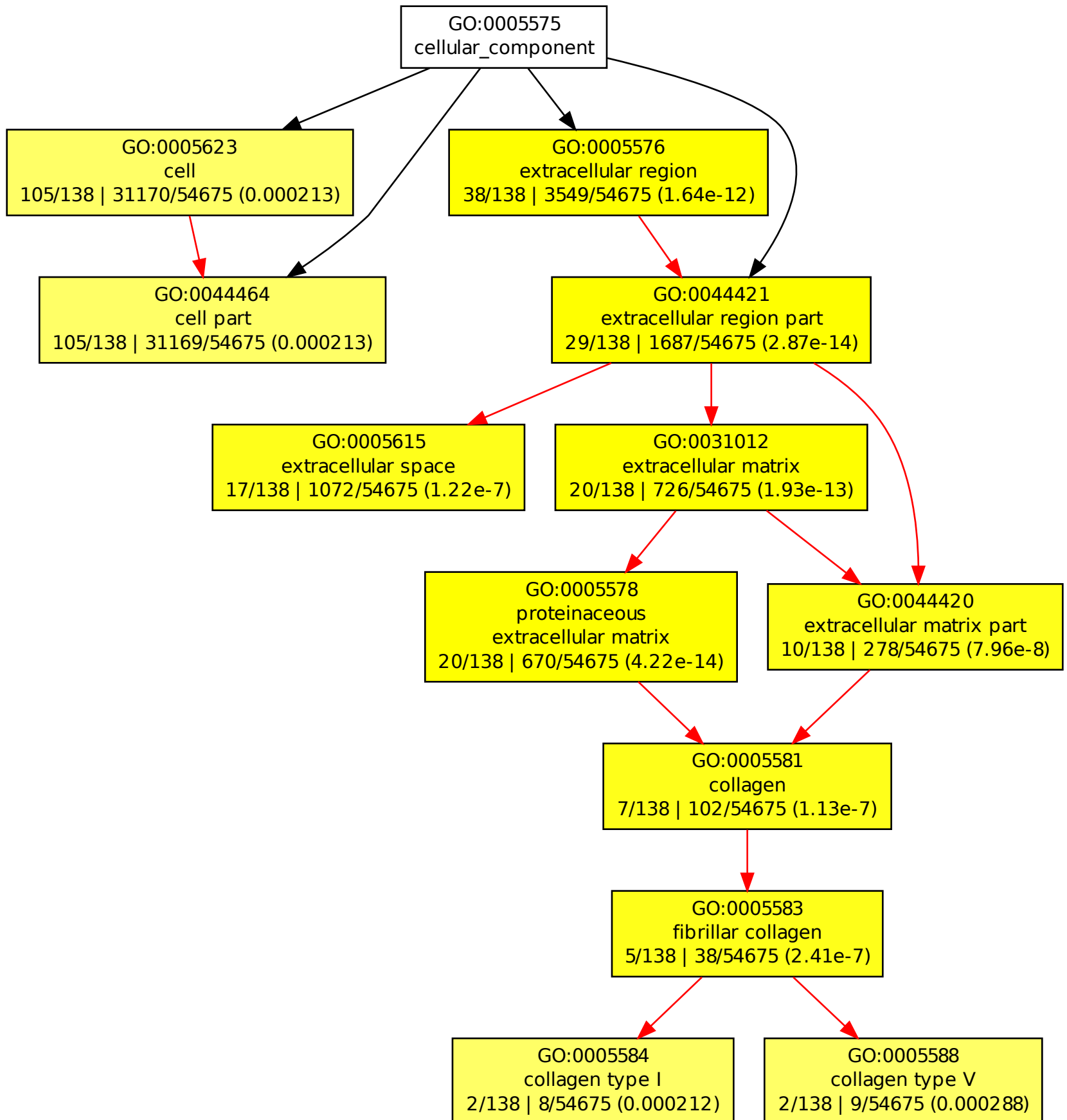

Supplement: Additional file 6 — GO cellular component. Graphical representation of GO annotation (cellular component). [file 1471-2407-11-437-S6.PDF]

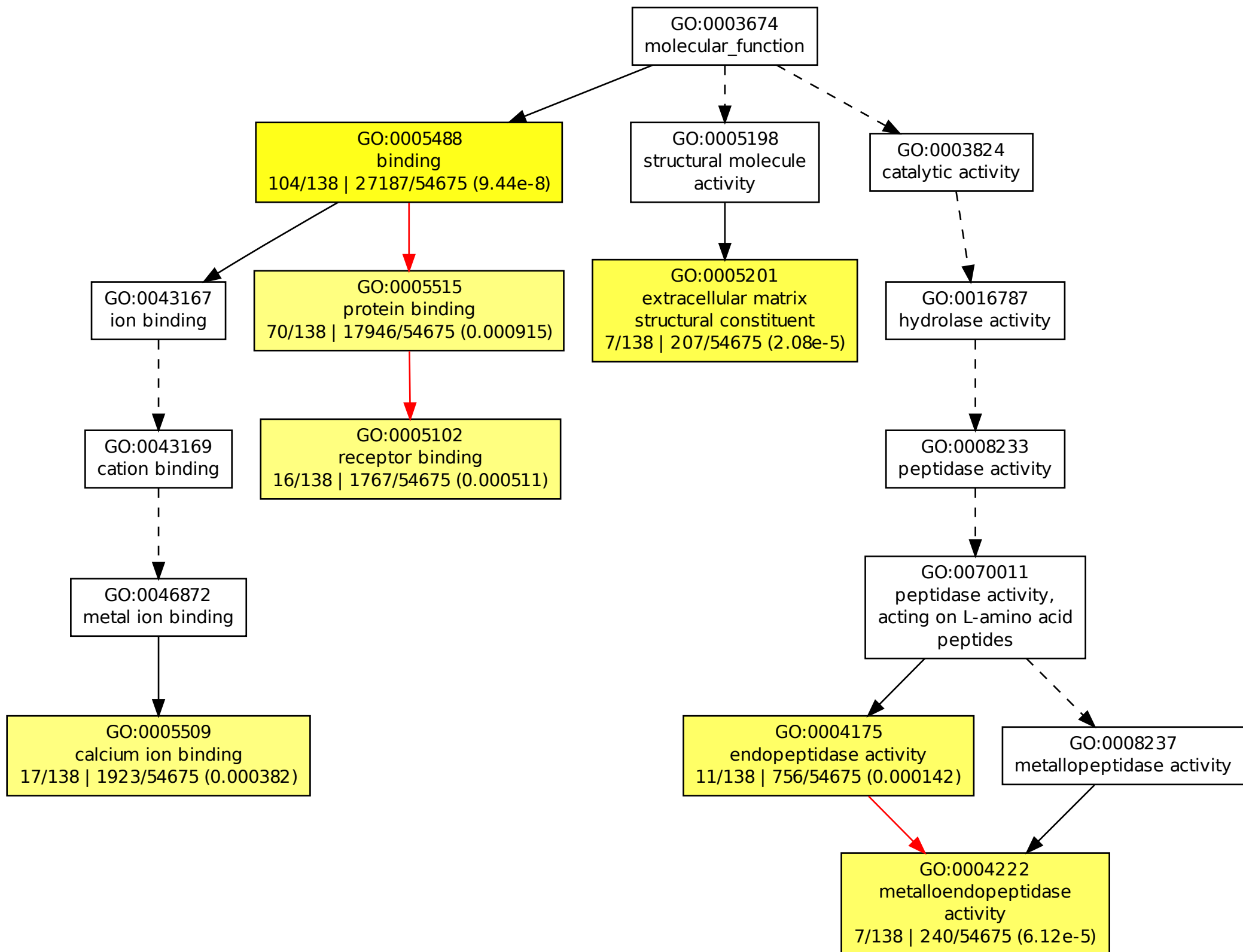

Supplement: Additional file 7 — GO molecular function. Graphical representation of GO annotation (molecular function). [file 1471-2407-11-437-S7.PDF]

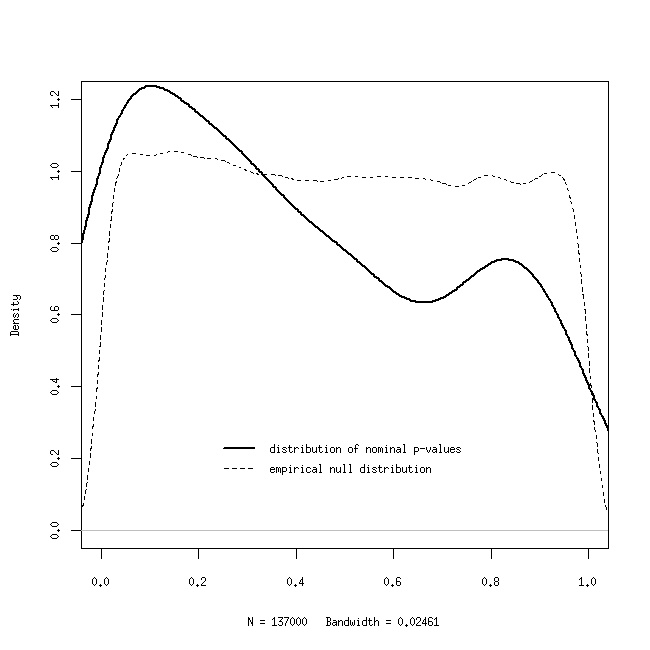

Supplement: Additional file 8 — Figure S1. Distribution of nominal p-values for univariate association of the 138 genes identified as over-expressed in OSCC with recurrence. P-values were determined by Cox regression using the maximum expression in any margin of each patient. The empirical null distribution was determined from association of these same genes with 1,000 permutations of the outcome labels. The observed nominal p-values are significantly enriched for small values (p = 0.001, Kolmogorov-Smirnov test). It is worth noting that recurrence was not used at any stage in the selection of these genes. [file 1471-2407-11-437-S8.PNG]

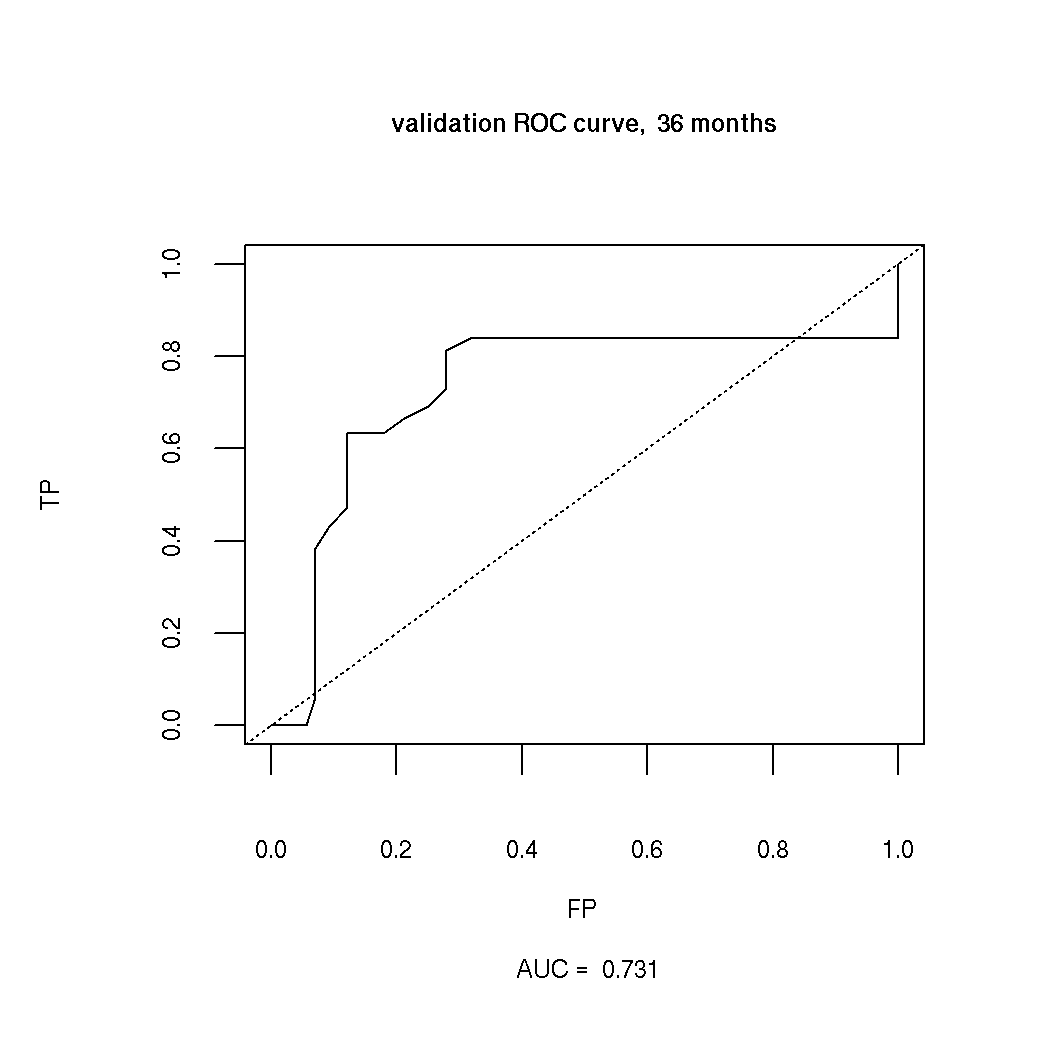

Supplement: Additional file 9 — Figure S2. Survival Receiver Operating Characteristic curve for recurrence at 36 months in the test set. Using a high threshold to define high-risk patients predicts a majority of recurrences (true positives) at a low false-positive rate (20%). While we maintained the standard median cutoff for this study due to the limited sample size, a larger study in the future may be able to further tune the cutoff threshold to optimize sensitivity and specificity in the context of the relative risks that treatment options informed by this prognostic score entail. The area under the ROC curve (AUC) for recurrence within 36 months is 0.73, which is an improvement over the expected AUC of 0.5 for non-predictive risk scores. [file 1471-2407-11-437-S9.PNG]

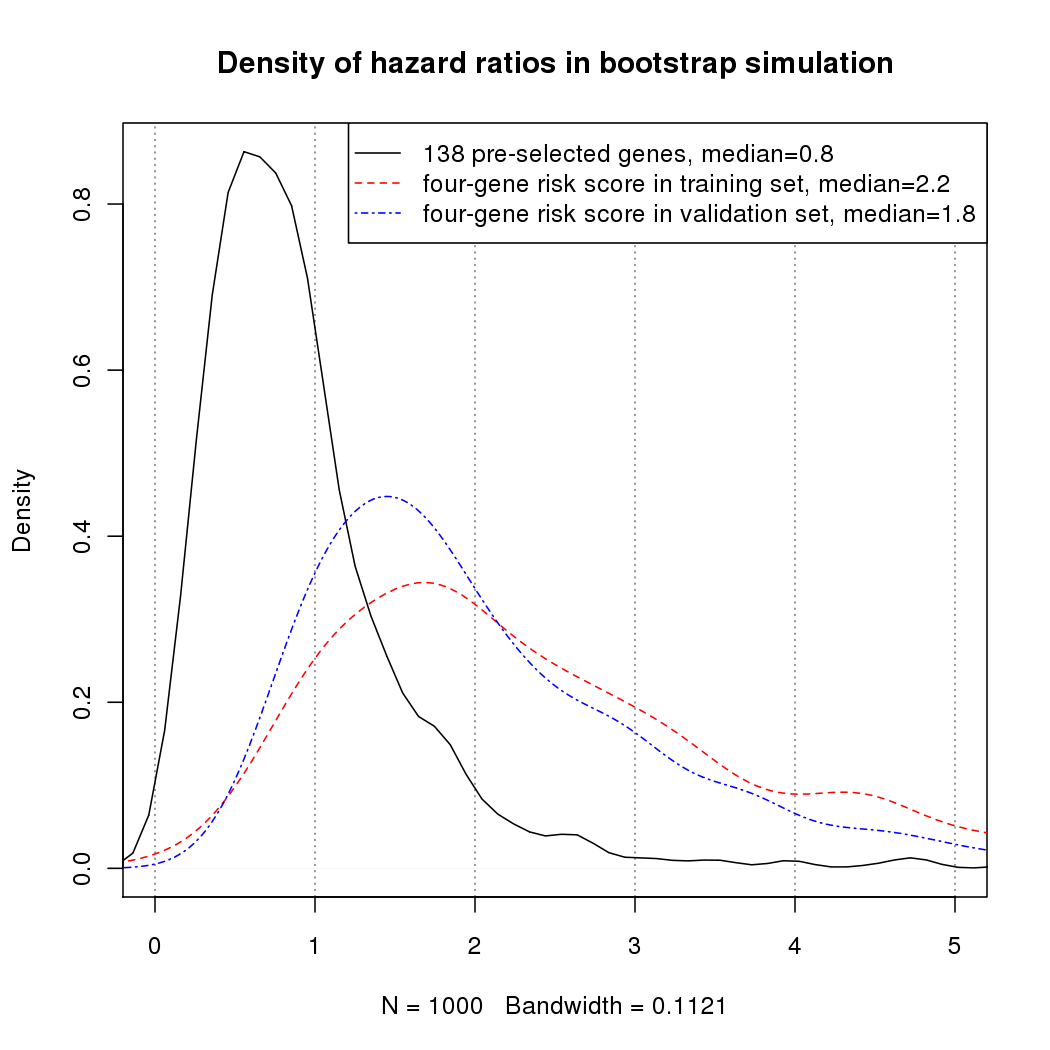

Supplement: Additional file 10 — Figure S3. Bootstrap validation of four-gene signature risk score in training and validation sets. Density lines represent the distribution of hazard ratios observed in 1,000 re-samplings of a single margin, randomly chosen, from each patient. [file 1471-2407-11-437-S10.PNG]
